# Finite-Resolution Information from Collision Statistics

**DOI:** 10.3390/e28080882

**Published:** 2026-08-05

**Authors:** Alexander J. Gates

**Affiliations:** School of Data Science, University of Virginia, Charlottesville, VA 22903, USA; agates@virginia.edu

**Keywords:** Shannon entropy, Rényi entropy, collision probability, mutual information, finite-sample estimation, power sums

## Abstract

Collision statistics provide a finite-resolution view of information by measuring how often independent samples fall on the same state and form the basis of integer-order Rényi entropies. Here, we use low-order Rényi entropies to characterize finite-resolution approximations to Shannon entropy and mutual information. Specifically, we determine what population information is captured by finite collision moments, we quantify how the resulting targets differ from their Shannon counterparts, and we analyze how accurately they can be estimated from finite samples. We use the interpolation remainder to identify structural approximation error induced by extrapolating from integer-order Rényi entropies to the Shannon point. We separate this deterministic error from finite-sample estimation error: increasing sample size improves estimation of a finite-resolution target but does not eliminate its deterministic difference from Shannon entropy or mutual information. Finally, we show that finite collision moments do not generally identify Shannon entropy, and that increasing collision order shifts sensitivity toward high-probability events. Our numerical experiments illustrate the approximation–estimation trade-off and evaluate collision-based approximations alongside plug-in and Miller–Madow estimators. Together, these results provide a principled way to use low-order coincidence structure as finite-resolution information, while making explicit what finite collision moments can and cannot reveal about Shannon entropy and mutual information.

## 1. Introduction

Estimating Shannon entropy and mutual information from finite discrete samples remains a persistent problem, especially when the alphabet is large relative to the number of observations. The empirical plug-in estimator of entropy is biased downward, while the plug-in estimator of mutual information is biased upward, with both effects becoming pronounced in sparse regimes [1,2,3]. Many estimators have been developed to address this problem, including Miller–Madow-type corrections [1], finite-sample bias corrections [4], Bayesian estimators such as NSB [5], and minimax-optimal estimators based on approximation theory [6,7,8]. These methods target Shannon entropy or mutual information directly.

Here, we take a complementary approach motivated by estimability. Rather than proposing another estimator of the Shannon functional, we formalize a family of finite-resolution information targets built from low-order collision moments. A collision moment measures the probability that two, three, or more independent samples take the same value. These probabilities are directly countable from data, have unbiased fixed-order estimators as *U*-statistics [9,10], and form the power sums underlying integer-order Rényi entropies [11]. Related work has studied the estimation of Rényi entropies from samples, including the behavior of collision-based estimators for fixed entropy orders [12]. Our focus is different: we use these finite-order quantities as observations of the Rényi entropy path in order to construct and analyze finite-resolution approximations to Shannon entropy and mutual information. Collision moments are also familiar in diversity measurement: the second-order collision probability is Simpson concentration, its complement is Simpson diversity, and its reciprocal is the order-two Hill number or effective number of types [13,14]. Thus collision statistics sit at the intersection of entropy estimation, Rényi entropy functionals, and count-based diversity measurement.

This perspective leads to a different question from classical Shannon estimation. Instead of asking how to recover H(P) or I(X;Y) as accurately as possible from finite samples, we ask: how much of Shannon entropy is visible from finitely many low-order collision moments? This distinction matters because two sources of error are often conflated. The first is statistical estimation error: how accurately can a collision moment be estimated from data? The second is approximation error: how much information is lost when the population target itself is restricted to finitely many collision moments? For fixed collision order, increasing the sample size reduces the first error but not the second. The order of the collision moments determines the finite-resolution population target, while the sample size determines how accurately that target is estimated.

The connection to Rényi entropy provides the organizing structure for this finite-resolution view. For a distribution $P=(p_{1},\ldots ,p_{m})$, the order-*k* collision probability

$$C_{k}\left(P\right)=\sum_{i}p_{i}^{k}$$

defines the integer-order Rényi entropy

$$H_{k}\left(P\right)=\frac{1}{1-k}\log C_{k}\left(P\right),$$

while Shannon entropy is recovered by the continuous limit k→1 [11,15]. We therefore view the sequence $H_{2}\left(P\right),H_{3}\left(P\right),\ldots$ as a set of finite-resolution observations of the Rényi entropy path away from the Shannon point. Low-order extrapolations from this path yield approximations to Shannon entropy, and applying the same construction to the marginal and joint distributions yields corresponding approximations to mutual information.

The primary contribution of this paper is a finite-resolution formulation of collision-based information, not a new universal estimator of Shannon entropy or mutual information. Many existing estimators target the Shannon functional directly and are evaluated by how accurately they recover that target from finite samples. Here, we instead treat a finite collection of collision moments as defining its own population-level information target. This shift makes explicit a distinction that is often implicit: the statistical problem of estimating collision moments from data is separate from the deterministic problem of approximating the Shannon limit from finitely many points on the Rényi entropy path. For fixed collision order, a collision-based estimator converges to a finite-resolution Rényi-based functional, not generally to Shannon entropy or mutual information. Changing the collision orders changes the target itself. Thus, the framework should be read as a way to characterize what is visible from low-order coincidence structure, when that information approximates Shannon quantities, and when irreducible information remains outside the finite moment representation.

The paper makes four contributions. First, we define the finite-resolution population targets induced by low-order collision moments and relate their approximation error to the classical interpolation remainder along the Rényi entropy path. Second, we extend the same construction to mutual information by applying the finite-resolution entropy approximation to marginal and joint distributions, using an additive Rényi entropy contrast whose Shannon limit is mutual information. Third, we give a fixed-order asymptotic analysis that separates estimation error from deterministic approximation error, showing that empirical collision estimators are centered on finite-resolution population targets rather than on Shannon quantities themselves. Fourth, we show that finite collision-moment truncations do not generally identify entropy, establishing an intrinsic limitation of any representation based only on finitely many low-order collisions. Our numerical experiments illustrate these approximation–estimation trade-offs and compare the resulting finite-resolution approximations with plug-in and Miller–Madow estimators.

The remainder of the paper is organized as follows. Section 2 introduces collision probabilities, Rényi entropy, and the corresponding entropy contrast for mutual information. Section 3 develops low-order approximations to Shannon entropy and mutual information. Section 4 separates estimation error from approximation error. Section 5 studies collision order as a concentration and resolution parameter. Section 6 proves that finite collision moments do not identify entropy. Section 7 presents numerical experiments, and Section 8 discusses implications for entropy estimation and finite-resolution information representations.

## 2. Preliminaries

Let $P=(p_{1},\ldots ,p_{m})$ be a discrete probability distribution on a finite alphabet X={1,…,m}. Throughout, we use logarithms in base *e*, so all entropies are measured in nats. The corresponding expressions in bits are obtained by dividing by log2.

### 2.1. Collision Probabilities

For α>0, define the order-α collision probability, or power sum, of *P* by

$$C_{\alpha }\left(P\right)=\sum_{i=1}^{m}p_{i}^{\alpha }.$$

When α=k is a positive integer, $C_{k}\left(P\right)$ has a direct sampling interpretation. If $X_{1},\ldots ,X_{k}$ are independent samples from *P* then

$$C_{k}\left(P\right)=\mathrm{Pr}\left\{X_{1}=X_{2}=\cdots =X_{k}\right\}.$$

Thus $C_{2}\left(P\right)$ is the probability of a pairwise collision, $C_{3}\left(P\right)$ is the probability of a triplet collision, and so on.

For a sample $X_{1},\ldots ,X_{N}$ from *P*, let $n_{i}$ denote the number of observations in state *i*. For fixed integer k≥2, the without-replacement empirical collision statistic is

C^k(P)=∑inikNk.

Equivalently,

C^k(P)=Nk−1∑1≤r1<⋯<rk≤Nδ{Xr1,⋯,Xrk}.

This is a *U*-statistic [9,10]. Moreover, it is unbiased for $C_{k}\left(P\right)$, since each unordered *k*-tuple has probability $C_{k}\left(P\right)$ of taking a common value:

E[C^k(P)]=Nk−1∑1≤r1<⋯<rk≤NPr{Xr1=⋯=Xrk}=Ck(P).

The fixed-order nature of this estimator will be important below: as *N* increases, ${\hat{C}}_{k}\left(P\right)$ estimates the same population quantity $C_{k}\left(P\right)$ more accurately, but the target itself remains fixed by the order *k*.

### 2.2. Rényi Entropy and the Shannon Limit

For α>0, α≠1, the Rényi entropy [11] of order α is

$$H_{\alpha }\left(P\right)=\frac{1}{1-\alpha }\log C_{\alpha }\left(P\right)=\frac{1}{1-\alpha }\log\sum_{i=1}^{m}p_{i}^{\alpha }.$$

The Shannon entropy is recovered by continuity as α→1 [15]:

$$H\left(P\right)=\lim_{\alpha \to 1}H_{\alpha }\left(P\right)=-\sum_{i=1}^{m}p_{i}\log p_{i}.$$

Indeed, since $C_{1}\left(P\right)=1$, applying l’Hôpital’s rule gives

$$\lim_{\alpha \to 1}\frac{\log C_{\alpha }\left(P\right)}{1-\alpha }=-{\left.\frac{d}{d\alpha }\log C_{\alpha }\left(P\right)\right|}_{\alpha =1}=-\sum_{i}p_{i}\log p_{i}.$$

Several special cases of the Rényi family are useful for interpretation. The order-two entropy

$$H_{2}\left(P\right)=-\log\sum_{i}p_{i}^{2}$$

is often called collision entropy because it is the negative logarithm of the probability that two independent draws coincide [16]. As α→∞, $H_{\alpha }\left(P\right)$ converges to the min-entropy,

$$H_{\infty }\left(P\right)=-\log\;{∥P∥}_{\infty }=-\log\max_{i}p_{i}.$$

Thus the Rényi parameter controls how strongly the entropy emphasizes high-probability states.

### 2.3. Joint Distributions and Mutual Information

Let *X* and *Y* be discrete random variables with joint distribution

$$P_{XY}={\left(p_{xy}\right)}_{x\in X,\;y\in Y}.$$

Let $P_{X}=\left(p_{x}\right)$ and $P_{Y}=\left(p_{y}\right)$ denote the marginals. The Shannon mutual information is

$$I\left(X;Y\right)=H\left(X\right)+H\left(Y\right)-H\left(X,Y\right)=\sum_{x,y}p_{xy}\log\frac{p_{xy}}{p_{x}p_{y}}.$$

For α>0, α≠1, define the marginal and joint collision probabilities

$$C_{\alpha }\left(X\right)=\sum_{x}p_{x}^{\alpha },\;C_{\alpha }\left(Y\right)=\sum_{y}p_{y}^{\alpha },\;C_{\alpha }\left(X,Y\right)=\sum_{x,y}p_{xy}^{\alpha }.$$

These define the corresponding Rényi entropies $H_{\alpha }\left(X\right)$, $H_{\alpha }\left(Y\right)$, and $H_{\alpha }\left(X,Y\right)$.

We define the Rényi entropy contrast:

$$K_{\alpha }\left(X;Y\right)=H_{\alpha }\left(X\right)+H_{\alpha }\left(Y\right)-H_{\alpha }\left(X,Y\right)=\frac{1}{1-\alpha }\left[\log\;C_{\alpha }\left(X\right)+\log\;C_{\alpha }\left(Y\right)-\log\;C_{\alpha }\left(X,Y\right)\right].$$

This contrast is symmetric in *X* and *Y* and invariant under relabeling of the states. It also has the Shannon mutual information as its order-one limit:

$$\lim_{\alpha \to 1}K_{\alpha }\left(X;Y\right)=I\left(X;Y\right).$$

We refer to $K_{\alpha }$ as an additive Rényi entropy contrast rather than as a Rényi mutual information. Several inequivalent Rényi generalizations of mutual information exist, with different axiomatic and operational properties [17,18,19]. The contrast used here is the direct analogue of the Shannon identity obtained by replacing Shannon entropies with Rényi entropies of common order α. It is useful for the present purpose because it defines a Rényi entropy-contrast path whose order-one limit is Shannon mutual information. However, for α≠1, $K_{\alpha }$ should not be interpreted as an operational or axiomatic Rényi mutual information: it need not be non-negative, and the condition $K_{\alpha }\left(X;Y\right)=0$ need not characterize independence. We use $K_{\alpha }$ only as an entropy-contrast path whose Shannon limit is MI; we do not require it to satisfy axioms proposed for Rényi mutual information.

## 3. Low-Order Collision Approximations to Entropy and Mutual Information

We now use the Rényi entropy path to construct finite-order approximations to Shannon entropy. These approximations should be understood as finite-resolution population functionals determined by a chosen set of collision orders, not as claims of uniformly optimal Shannon estimation. The key idea is to treat the integer-order values $H_{2}\left(P\right),H_{3}\left(P\right),H_{4}\left(P\right),\ldots$ as observations of the function $h\left(\alpha \right)=H_{\alpha }\left(P\right)$ away from the Shannon point α=1. Low-order approximations to H(P)=h(1) and their errors are then obtained by extrapolating this path back to α=1.

### 3.1. Approximating Shannon Entropy

Let $P=(p_{1},\ldots ,p_{m})$ be a discrete distribution and define $h\left(\alpha \right)=H_{\alpha }\left(P\right).$ Since h(1)=H(P), we approximate Shannon entropy by interpolating *h* at integer orders α=2,…,r+1 and evaluating the interpolating polynomial at α=1.

For an integer r≥1, let $L_{r-1}\left(\alpha \right)$ be the unique polynomial of degree at most r−1 satisfying

$$L_{r-1}\left(j+1\right)=H_{j+1}\left(P\right),\;j=1,\ldots ,r.$$

We define the order-*r* collision approximation to Shannon entropy by ${\tilde{H}}_{r}\left(P\right)=L_{r-1}\left(1\right)$.

Using the Lagrange form of the interpolating polynomial, this approximation can be written explicitly as

H˜r(P)=∑j=1r(−1)j−1rjHj+1(P)=−∑j=1r(−1)j−1rj1j log Cj+1(P).

Thus the first few approximations are

$${\tilde{H}}_{1}\left(P\right)=H_{2}\left(P\right),$$

$${\tilde{H}}_{2}\left(P\right)=2H_{2}\left(P\right)-H_{3}\left(P\right)=-2\log C_{2}\left(P\right)+\frac{1}{2}\log C_{3}\left(P\right),$$

and

$${\tilde{H}}_{3}\left(P\right)=3H_{2}\left(P\right)-3H_{3}\left(P\right)+H_{4}\left(P\right)=-3\log C_{2}\left(P\right)+\frac{3}{2}\log C_{3}\left(P\right)-\frac{1}{3}\log C_{4}\left(P\right).$$

The subscript *r* indicates the number of integer Rényi entropies used in the approximation. Equivalently, ${\tilde{H}}_{r}$ uses collision probabilities up to order r+1.

### 3.2. Approximation Error

The approximation error is deterministic and depends on the regularity of the Rényi entropy path near α=1. The following result is an application of the classical Lagrange interpolation remainder. It should be read as a structural identity for the population approximation error, not as a data-driven error certificate.

**Theorem 1** (Interpolation remainder from low-order collision entropies)**.**
*Let P be a discrete distribution and suppose that $h\left(\alpha \right)=H_{\alpha }\left(P\right)$ is r times continuously differentiable on [1,r+1]. Then there exists ξ∈(1,r+1) such that*

$$H\left(P\right)-{\tilde{H}}_{r}\left(P\right)={\left(-1\right)}^{r}h^{\left(r\right)}\left(\xi \right).$$

*Consequently,*

$$\left|H\left(P\right)-{\tilde{H}}_{r}\left(P\right)\right|\leq \underset{\alpha \in [1,r+1]}{\sup}\left|h^{\left(r\right)}\left(\alpha \right)\right|.$$

**Proof.**  Let $L_{r-1}$ be the degree-(r−1) interpolating polynomial for *h* at the points 2,3,…,r+1. The interpolation remainder gives

$$h\left(1\right)-L_{r-1}\left(1\right)=\frac{h^{\left(r\right)}\left(\xi \right)}{r!}\prod_{j=1}^{r}\left(1-(j+1)\right)$$

for some ξ∈(1,r+1). Since

$$\prod_{j=1}^{r}\left(1-(j+1)\right)=\prod_{j=1}^{r}\left(-j\right)={\left(-1\right)}^{r}r!,$$

we obtain

$$h\left(1\right)-L_{r-1}\left(1\right)={\left(-1\right)}^{r}h^{\left(r\right)}\left(\xi \right).$$

Using h(1)=H(P) and $L_{r-1}\left(1\right)={\tilde{H}}_{r}\left(P\right)$ proves the stated identity. Taking absolute values gives the bound. □

For the pair–triplet approximation, ${\tilde{H}}_{2}\left(P\right)=2H_{2}\left(P\right)-H_{3}\left(P\right)$, Theorem 1 gives

$$\left|H\left(P\right)-{\tilde{H}}_{2}\left(P\right)\right|\leq \underset{\alpha \in [1,3]}{\sup}\left|h^{\prime \prime }\left(\alpha \right)\right|.$$

Likewise, for the pair–triplet–quartet approximation, ${\tilde{H}}_{3}\left(P\right)=3H_{2}\left(P\right)-3H_{3}\left(P\right)+H_{4}\left(P\right)$, we obtain

$$\left|H\left(P\right)-{\tilde{H}}_{3}\left(P\right)\right|\leq \underset{\alpha \in [1,4]}{\sup}\left|h^{\left(3\right)}\left(\alpha \right)\right|.$$

Theorem 1 gives a geometric interpretation of the approximation. The quantities $H_{2}\left(P\right),H_{3}\left(P\right),\ldots$$H_{r+1}\left(P\right)$ are values of the Rényi entropy path away from the Shannon point α=1. The approximation ${\tilde{H}}_{r}\left(P\right)$ extrapolates this path back to α=1 using a polynomial fitted to those integer-order values. The error is therefore controlled by how much the Rényi entropy path bends over the interval [1,r+1]. When the path is nearly polynomial of degree at most r−1 on this interval, the approximation is accurate; when the path has substantial higher-order variation, the extrapolation can retain non-negligible deterministic error.

Theorem 1 should be interpreted as a structural statement about the source of approximation error, rather than an operational or distribution-free guarantee. In particular, the derivative term $\sup_{\alpha \in [1,r+1]}\left|h^{\left(r\right)}\left(\alpha \right)\right|$ is not determined by the finite collision moments $C_{2}\left(P\right),\ldots ,C_{r+1}\left(P\right)$ used in the approximation, and it is not uniformly bounded over all discrete distributions without additional assumptions. Sharper or computable guarantees would require further conditions, such as bounded support with controlled minimum mass, specified tail behavior, or regularity assumptions on the Rényi entropy path. The error in Theorem 1 is therefore not a finite-sample effect: it is a deterministic property of the population distribution and of the finite set of collision orders used in the approximation. Increasing the sample size can reduce estimation error for the collision probabilities, but it cannot remove this interpolation error unless additional collision orders are incorporated or the approximation scheme is changed.

### 3.3. Extension to Approximate Mutual Information

The extension to mutual information is direct. Let *X* and *Y* be discrete random variables with joint distribution $P_{XY}$ and marginals $P_{X}$ and $P_{Y}$. Define

$${\tilde{I}}_{r}\left(X;Y\right)={\tilde{H}}_{r}\left(X\right)+{\tilde{H}}_{r}\left(Y\right)-{\tilde{H}}_{r}\left(X,Y\right).$$

Using the definition of ${\tilde{H}}_{r}$, this can be written as

I˜r(X;Y)=∑j=1r(−1)j−1rjHj+1(X)+Hj+1(Y)−Hj+1(X,Y).

Therefore,

I˜r(X;Y)=∑j=1r(−1)j−1rjKj+1(X;Y),

where

$$K_{\alpha }\left(X;Y\right)=H_{\alpha }\left(X\right)+H_{\alpha }\left(Y\right)-H_{\alpha }\left(X,Y\right)$$

is the Rényi entropy contrast introduced in Section 2.

The first few mutual-information approximations are

$${\tilde{I}}_{1}\left(X;Y\right)=K_{2}\left(X;Y\right),$$

$${\tilde{I}}_{2}\left(X;Y\right)=2K_{2}\left(X;Y\right)-K_{3}\left(X;Y\right),$$

and

$${\tilde{I}}_{3}\left(X;Y\right)=3K_{2}\left(X;Y\right)-3K_{3}\left(X;Y\right)+K_{4}\left(X;Y\right).$$

These use only low-order marginal and joint collision probabilities.

The interpolation theorem for mutual information follows immediately from the entropy result, or equivalently by applying the same interpolation argument to the Rényi contrast path.

**Corollary 1** (Interpolation remainder for low-order collision mutual information)**.**
*Let $g\left(\alpha \right)=K_{\alpha }\left(X;Y\right)$ and suppose that g is r times continuously differentiable on [1,r+1]. Then there exists ξ∈(1,r+1) such that*

$$I\left(X;Y\right)-{\tilde{I}}_{r}\left(X;Y\right)={\left(-1\right)}^{r}g^{\left(r\right)}\left(\xi \right).$$

*Consequently,*

$$|I\left(X;Y\right)-{\tilde{I}}_{r}\left(X;Y\right)|\leq \underset{\alpha \in [1,r+1]}{\sup}|g^{\left(r\right)}\left(\alpha \right)|.$$

**Proof.**  Apply Theorem 1 to the contrast path

$$g\left(\alpha \right)=K_{\alpha }\left(X;Y\right).$$

Since g(1)=I(X;Y) and since ${\tilde{I}}_{r}\left(X;Y\right)$ is the value at α=1 of the degree-(r−1) polynomial interpolating *g* at α=2,…,r+1, the same interpolation remainder gives the result. □

As in the entropy case, Corollary 1 is a structural population statement. The derivative term of the contrast path $g\left(\alpha \right)=K_{\alpha }\left(X;Y\right)$ is not determined by the finite collection of marginal and joint collision probabilities used to form ${\tilde{I}}_{r}\left(X;Y\right)$. Thus the corollary identifies the source of deterministic approximation error, but it does not provide a distribution-free or directly computable error certificate for a fixed data set.

Thus the mutual-information approximation is not a separate construction. It is the entropy approximation applied to the marginal and joint distributions. The population quantity ${\tilde{I}}_{r}\left(X;Y\right)$ depends only on the finite collection of collision probabilities

$$\{C_{k}\left(X\right),C_{k}\left(Y\right),C_{k}\left(X,Y\right):k=2,\ldots ,r+1\}.$$

In collision-probability form,

$$K_{k}\left(X;Y\right)=\frac{1}{1-k}\left[\log C_{k}\left(X\right)+\log C_{k}\left(Y\right)-\log C_{k}\left(X,Y\right)\right],$$

so

I˜r(X;Y)=−∑j=1r(−1)j−1rj1jlogCj+1(X)+logCj+1(Y)−logCj+1(X,Y).

This expression makes explicit that ${\tilde{I}}_{r}\left(X;Y\right)$ uses low-order coincidence information from the two marginals and the joint distribution.

### 3.4. Independence Baseline

The collision-based mutual-information approximations inherit the independence baseline of mutual information at the population level. If *X* and *Y* are independent then

$$p_{xy}=p_{x}p_{y}.$$

Therefore, for every α>0,

$$C_{\alpha }\left(X,Y\right)=\sum_{x,y}{\left(p_{x}p_{y}\right)}^{\alpha }=\left(\sum_{x}p_{x}^{\alpha }\right)\left(\sum_{y}p_{y}^{\alpha }\right)=C_{\alpha }\left(X\right)C_{\alpha }\left(Y\right).$$

It follows that

$$K_{\alpha }\left(X;Y\right)=0$$

for every α≠1, and hence

$${\tilde{I}}_{r}\left(X;Y\right)=0$$

for every approximation order *r*.

This establishes a one-way independence baseline. If *X* and *Y* are independent then $K_{\alpha }\left(X;Y\right)=0$ for every α≠1, and therefore ${\tilde{I}}_{r}\left(X;Y\right)=0$ for every approximation order *r*. The converse should not be assumed. For α≠1, the additive Rényi entropy contrast $K_{\alpha }$ is not a full dependence measure in the axiomatic or operational sense: it may be negative, and zero contrast need not imply independence. Thus, in this paper, $K_{\alpha }$ is used only because it connects low-order marginal and joint collision entropies to the Shannon mutual information limit.

For fixed *r*, ${\tilde{I}}_{r}\left(X;Y\right)$ is therefore a finite-resolution dependence functional constructed from low-order collision information. It is calibrated to the independence baseline in the sense that independence implies ${\tilde{I}}_{r}\left(X;Y\right)=0$, and it approximates Shannon mutual information when the Rényi contrast path is sufficiently regular near α=1.

### 3.5. Finite Moment Truncation

The finite-order nature of ${\tilde{H}}_{r}$ and ${\tilde{I}}_{r}$ is both a strength and a limitation. It is a strength because the required ingredients are low-order collision probabilities, which are directly estimable from coincidence counts. It is a limitation because finitely many collision probabilities do not generally determine Shannon entropy.

For a distribution on *m* symbols, sufficiently many power sums determine the distribution up to permutation. However, a finite truncation $C_{2}\left(P\right),\ldots ,C_{r+1}\left(P\right)$ does not generally identify *P* when the alphabet is large enough. Consequently, it cannot generally determine H(P). We return to this point in Section 6, where we prove a non-identifiability result for finite collision-moment truncations. The purpose of the low-order approximation is therefore not to eliminate the need for Shannon entropy estimators. Rather, it provides a controlled way to ask what part of Shannon entropy and mutual information is visible from a finite number of collision statistics, how that information changes as additional collision orders are included, and how estimation error differs from approximation error.

## 4. Fixed-Order Asymptotics

The preceding section defined population approximations ${\tilde{H}}_{r}\left(P\right)$ and ${\tilde{I}}_{r}\left(X;Y\right)$ from finitely many collision orders. We now consider their empirical counterparts. The main point is that for fixed approximation order, increasing the sample size improves estimation of the finite-resolution target but it does not remove the deterministic approximation error relative to Shannon entropy or mutual information.

### 4.1. Empirical Collision Entropies

Let $X_{1},\ldots ,X_{N}$ be i.i.d. samples from a discrete distribution *P*. For fixed integer k≥2, let

C^k(P)=∑inikNk

be the empirical collision probability. The corresponding empirical collision entropy is

$${\hat{H}}_{k}\left(P\right)=\frac{1}{1-k}\log{\hat{C}}_{k}\left(P\right),$$

whenever ${\hat{C}}_{k}\left(P\right)>0$.

For a fixed approximation order *r*, define the empirical low-order entropy approximation by

H˜^r(P)=∑j=1r(−1)j−1rjH^j+1(P).

Equivalently,

H˜^r(P)=−∑j=1r(−1)j−1rj1j log C^j+1(P).

This estimator uses empirical collision probabilities up to order r+1.

Since ${\hat{C}}_{k}\left(P\right)$ is an unbiased and consistent estimator of $C_{k}\left(P\right)$ for fixed *k*, the continuous mapping theorem gives

$${\hat{H}}_{k}\left(P\right)\overset{p}{\to }H_{k}\left(P\right)$$

provided $C_{k}\left(P\right)>0$. Therefore,

$${\hat{\tilde{H}}}_{r}\left(P\right)\overset{p}{\to }{\tilde{H}}_{r}\left(P\right)$$

for fixed *r* as N→∞.

Thus ${\hat{\tilde{H}}}_{r}\left(P\right)$ is consistent for the finite-order approximation ${\tilde{H}}_{r}\left(P\right)$, not necessarily for the Shannon entropy H(P). This gives the decomposition

$${\hat{\tilde{H}}}_{r}\left(P\right)-H\left(P\right)=\underset{\mathrm{estimation}\;\mathrm{error}}{\underset{︸}{{\hat{\tilde{H}}}_{r}\left(P\right)-{\tilde{H}}_{r}\left(P\right)}}+\underset{\mathrm{approximation}\;\mathrm{error}}{\underset{︸}{{\tilde{H}}_{r}\left(P\right)-H\left(P\right)}}.$$

The first term vanishes in probability as N→∞ for fixed *r*. The second term is the interpolation error studied in Theorem 1 and remains fixed for a given population distribution and approximation order.

### 4.2. Asymptotic Behavior for Fixed Collision Orders

The fixed-order collision statistics are bounded *U*-statistics, but their asymptotic behavior depends on whether their first Hoeffding projections are nonzero. We therefore state the projection and covariance structure explicitly; derivations are given in Appendix A.1.

For fixed k≥2, the empirical collision probability ${\hat{C}}_{k}\left(P\right)$ is a bounded *U*-statistic with kernel $h_{k}\left(x_{1},\ldots ,x_{k}\right)=1\left\{x_{1}=\cdots =x_{k}\right\}$. Its first Hoeffding projection is $\psi_{k}\left(x\right)=p_{x}^{k-1}-C_{k}\left(P\right)$. Therefore, for collision orders *k* and *ℓ*,

$$E\;\left[\psi_{k}\left(X\right)\psi_{\ell }\left(X\right)\right]=C_{k+\ell -1}\left(P\right)-C_{k}\left(P\right)C_{\ell }\left(P\right).$$

Let ${\hat{C}}_{r}={\left({\hat{C}}_{2},\ldots ,{\hat{C}}_{r+1}\right)}^{T}$ and $C_{r}={\left(C_{2},\ldots ,C_{r+1}\right)}^{T}$. The multivariate *U*-statistic central limit theorem gives

(1) $$\sqrt{N}\left({\hat{C}}_{r}-C_{r}\right)\Rightarrow N\;\left(0,\Gamma_{r}^{\left(C\right)}\right),\;{\left[\Gamma_{r}^{\left(C\right)}\right]}_{k\ell }=k\ell \left[C_{k+\ell -1}\left(P\right)-C_{k}\left(P\right)C_{\ell }\left(P\right)\right],$$

for k,ℓ∈{2,…,r+1}. In particular,

$${\left[\Gamma_{r}^{\left(C\right)}\right]}_{kk}=k^{2}\mathrm{Var}\;\left(p_{X}^{k-1}\right).$$

Thus the order-*k* collision statistic is first-order nondegenerate precisely when $p_{X}^{k-1}$ is not constant almost surely. For a finite discrete distribution, this condition fails when *P* is uniform on its positive-probability support.

Now let ${\hat{H}}_{r}={\left({\hat{H}}_{2},\ldots ,{\hat{H}}_{r+1}\right)}^{T}$ and $H_{r}={\left(H_{2},\ldots ,H_{r+1}\right)}^{T}$. Since the derivative of $H_{k}={\left(1-k\right)}^{-1}\log C_{k}$ with respect to $C_{k}$ is ${\left\{\left(1-k\right)C_{k}\right\}}^{-1}$, the multivariate delta method gives

(2) $$\sqrt{N}\left({\hat{H}}_{r}-H_{r}\right)\Rightarrow N\;\left(0,\Gamma_{r}^{\left(H\right)}\right),\;{\left[\Gamma_{r}^{\left(H\right)}\right]}_{k\ell }=\frac{k\ell \left[C_{k+\ell -1}\left(P\right)-C_{k}\left(P\right)C_{\ell }\left(P\right)\right]}{\left(1-k\right)\left(1-\ell \right)C_{k}\left(P\right)C_{\ell }\left(P\right)}.$$

Define ar,k=(−1)k−2rk−1 for k=2,…,r+1, so that

$${\tilde{H}}_{r}\left(P\right)=\sum_{k=2}^{r+1}a_{r,k}H_{k}\left(P\right),\;{\hat{\tilde{H}}}_{r}\left(P\right)=\sum_{k=2}^{r+1}a_{r,k}{\hat{H}}_{k}\left(P\right).$$

Let $a_{r}={\left(a_{r,2},\ldots ,a_{r,r+1}\right)}^{T}$. Then

(3) $$\sqrt{N}\left[{\hat{\tilde{H}}}_{r}\left(P\right)-{\tilde{H}}_{r}\left(P\right)\right]\Rightarrow N\;\left(0,\tau_{H,r}^{2}\right),\;\tau_{H,r}^{2}=a_{r}^{T}\Gamma_{r}^{\left(H\right)}a_{r},$$

provided $\tau_{H,r}^{2}>0$. Thus, the Gaussian limit in Equation (3) describes sampling fluctuations around the finite-resolution population target ${\tilde{H}}_{r}\left(P\right)$, not around the Shannon entropy H(P). The cross-order covariance terms are necessary because all collision statistics are computed from the same sample.

Combining this result with the deterministic interpolation error gives

(4) $${\hat{\tilde{H}}}_{r}\left(P\right)-H\left(P\right)=\left[{\tilde{H}}_{r}\left(P\right)-H\left(P\right)\right]+O_{p}\;\left(N^{-1/2}\right)$$

whenever the first-order asymptotic variance is positive.

Uniform distributions require separate treatment. If *P* is uniform on *m* states then $p_{x}=1/m$, $C_{k}\left(P\right)=m^{1-k}$, and $\psi_{k}\left(x\right)=0$ for every *x*. The $N^{1/2}$-scaled Gaussian limit in Equation (1) is therefore degenerate. As shown in Appendix A.2, the leading term is governed by the second Hoeffding projection and has an *N*-scaled, non-Gaussian limit. Distributions close to uniformity may have small first-order variances, so the first-order Gaussian approximation may require relatively large samples.

### 4.3. Extension of Asymptotic Behavior to Mutual Information

The same distinction applies to the mutual-information approximation, but the marginal and joint collision statistics must be treated jointly because they are computed from the same paired sample. Let Z=(X,Y), and for A∈{X,Y,XY} define

$$\psi_{A,k}\left(Z\right)=\left\{\begin{array}{cc} p_{X}{\left(X\right)}^{k-1}-C_{k}\left(X\right), & A=X,\\ p_{Y}{\left(Y\right)}^{k-1}-C_{k}\left(Y\right), & A=Y,\\ p_{XY}{\left(X,Y\right)}^{k-1}-C_{k}\left(X,Y\right), & A=XY. \end{array}\right.$$

For A,B∈{X,Y,XY} and k,ℓ∈{2,…,r+1}, the joint asymptotic covariance matrix of the collision estimators has entries

(5) $${\left[\Gamma_{r}^{\left(XY\right)}\right]}_{\left(A,k),(B,\ell \right)}=k\ell \;E\;\left[\psi_{A,k}\left(Z\right)\psi_{B,\ell }\left(Z\right)\right].$$

Appendix A.3 gives the corresponding projection calculation.

Let $s_{X}=s_{Y}=1$ and $s_{XY}=-1$, and define

$$w_{A,k}=\frac{s_{A}a_{r,k}}{\left(1-k\right)C_{k}\left(A\right)}.$$

Collecting these coefficients into a vector $w_{r}$, the multivariate delta method gives

(6) $$\sqrt{N}\left[{\hat{\tilde{I}}}_{r}\left(X;Y\right)-{\tilde{I}}_{r}\left(X;Y\right)\right]\Rightarrow N\;\left(0,\tau_{I,r}^{2}\right),\;\tau_{I,r}^{2}=w_{r}^{T}\Gamma_{r}^{\left(XY\right)}w_{r},$$

provided $\tau_{I,r}^{2}>0$. Thus, the Gaussian limit in Equation (6) describes sampling fluctuations around the finite-resolution dependence target ${\tilde{I}}_{r}\left(X;Y\right)$, not around the Shannon mutual information I(X;Y).

Consequently,

(7) $${\hat{\tilde{I}}}_{r}\left(X;Y\right)-I\left(X;Y\right)=\left[{\tilde{I}}_{r}\left(X;Y\right)-I\left(X;Y\right)\right]+O_{p}\;\left(N^{-1/2}\right)$$

in the nondegenerate case. If $\tau_{I,r}^{2}=0$ then the $N^{1/2}$-scaled Gaussian limit is degenerate and higher-order Hoeffding projections determine the leading asymptotic behavior.

### 4.4. Interpretation of Asymptotic Behavior

The fixed-order asymptotic behavior clarifies the role of sample size. Increasing *N* makes empirical collision probabilities more accurate estimates of their population counterparts. It does not make a fixed collision order more Shannon-like. For example,

$${\hat{\tilde{I}}}_{2}\left(X;Y\right)=2{\hat{K}}_{2}\left(X;Y\right)-{\hat{K}}_{3}\left(X;Y\right)$$

converges to

$${\tilde{I}}_{2}\left(X;Y\right)=2K_{2}\left(X;Y\right)-K_{3}\left(X;Y\right),$$

not necessarily to I(X;Y).

Consequently, a fixed-order collision approximation can exhibit a persistent difference from Shannon entropy or mutual information even when the sample size is large. This persistent difference is not a failure of estimation; it is the deterministic approximation error associated with using finitely many collision orders. The sample size controls statistical precision, while the collision orders determine the population target.

## 5. Collision Order as a Concentration and Resolution Parameter

The collision order does more than index a family of estimators. It also changes which parts of the distribution are emphasized. Low orders aggregate probability mass broadly across the support, while high orders increasingly concentrate on the largest probabilities. This section makes that statement precise and interprets the order *k* as a resolution parameter.

Let $P=(p_{1},\ldots ,p_{m})$ be a discrete distribution, with probabilities ordered as

$$p_{1}\geq p_{2}\geq \cdots \geq p_{m}\geq 0.$$

Recall that the order-*k* collision probability is

$$C_{k}\left(P\right)=\sum_{i=1}^{m}p_{i}^{k}.$$

For larger *k*, the terms $p_{i}^{k}$ magnify differences among probabilities: high-probability states remain visible, while lower-probability states are suppressed.

### 5.1. Convergence Toward the Largest Probability

The following elementary result shows that the collision probability becomes dominated by the largest mass as the order increases.

**Lemma 1** (Collision roots converge to the maximum mass)**.**
*Let $P=(p_{1},\ldots ,p_{m})$ be a discrete distribution and let*

$$p_{\max}=\max_{i}p_{i}.$$

*Then*

$$\lim_{k\to \infty }C_{k}{\left(P\right)}^{1/k}=p_{\max}.$$

**Proof.**  Since $p_{\max}^{k}$ is one term in the sum defining $C_{k}\left(P\right)$,

$$p_{\max}^{k}\leq C_{k}\left(P\right).$$

Also, since each $p_{i}\leq p_{\max}$,

$$C_{k}\left(P\right)=\sum_{i}p_{i}^{k}\leq mp_{\max}^{k}.$$

Taking *k*-th roots gives

$$p_{\max}\leq C_{k}{\left(P\right)}^{1/k}\leq m^{1/k}p_{\max}.$$

Since $m^{1/k}\to 1$ as k→∞, the result follows. □

This result is the collision-probability form of the standard convergence of Rényi entropy to min-entropy:

$$H_{k}\left(P\right)=\frac{1}{1-k}\log C_{k}\left(P\right)⟶-\log p_{\max}=H_{\infty }\left(P\right).$$

Thus, increasing *k* moves the entropy functional away from the Shannon regime and toward a quantity determined by the largest atom of the distribution.

### 5.2. Tail Suppression

The preceding lemma describes the limiting behavior as k→∞. The next bound quantifies how quickly lower-probability states lose influence.

For s<m, define the top-*s* contribution to the order-*k* collision probability as

$$R_{s}\left(k\right)=\frac{\sum_{i=1}^{s}p_{i}^{k}}{\sum_{i=1}^{m}p_{i}^{k}}.$$

Thus $R_{s}\left(k\right)$ is the fraction of the collision probability contributed by the *s* largest states.

**Lemma 2** (Exponential suppression of lower-probability states)**.**
*Let $P=(p_{1},\ldots ,p_{m})$ be ordered so that*

$$p_{1}\geq p_{2}\geq \cdots \geq p_{m}.$$

*For any s<m with $p_{s}>0$,*

$$1-R_{s}\left(k\right)\leq \frac{m-s}{s}{\left(\frac{p_{s+1}}{p_{s}}\right)}^{k}.$$

*In particular, if $p_{s}>p_{s+1}$ then the contribution of the tail beyond the top s states decays exponentially in k.*

**Proof.**  We have

$$1-R_{s}\left(k\right)=\frac{\sum_{i=s+1}^{m}p_{i}^{k}}{\sum_{i=1}^{m}p_{i}^{k}}.$$

Since the denominator is at least the contribution of the top *s* states,

$$1-R_{s}\left(k\right)\leq \frac{\sum_{i=s+1}^{m}p_{i}^{k}}{\sum_{i=1}^{s}p_{i}^{k}}.$$

For i>s, $p_{i}\leq p_{s+1}$, so

$$\sum_{i=s+1}^{m}p_{i}^{k}\leq \left(m-s\right)p_{s+1}^{k}.$$

For i≤s, $p_{i}\geq p_{s}$, so

$$\sum_{i=1}^{s}p_{i}^{k}\geq sp_{s}^{k}.$$

Combining the two inequalities gives

$$1-R_{s}\left(k\right)\leq \frac{m-s}{s}{\left(\frac{p_{s+1}}{p_{s}}\right)}^{k}.$$

If $p_{s}>p_{s+1}$ then $p_{s+1}/p_{s}<1$, so the bound decays exponentially in *k*. □

Lemma 2 provides a finite-order version of the concentration interpretation. The order *k* determines how sharply the collision probability distinguishes high-mass states from lower-mass states. When the leading probabilities are separated from the tail, increasing *k* rapidly concentrates the collision statistic on those leading states.

### 5.3. Effective Support at Order k

The concentration behavior can also be expressed through the effective support associated with Rényi entropy. Define

$$S_{k}\left(P\right)=\exp\left(H_{k}\left(P\right)\right).$$

For k≠1,

$$S_{k}\left(P\right)=C_{k}{\left(P\right)}^{1/(1-k)}.$$

This quantity is often interpreted as an effective number of states at order *k*. At k=1, it reduces by continuity to the Shannon effective support,

$$S_{1}\left(P\right)=\exp\left(H\left(P\right)\right).$$

At k=2,

$$S_{2}\left(P\right)=\frac{1}{\sum_{i}p_{i}^{2}},$$

the reciprocal of the pair-collision probability. As k→∞,

$$S_{k}\left(P\right)\to \frac{1}{p_{\max}}.$$

Thus increasing *k* decreases the effective support from a Shannon-weighted notion of diversity toward a support size determined by the largest probability. This gives another interpretation of collision order as a resolution parameter: larger values of *k* resolve the distribution at a scale dominated by its largest atoms.

### 5.4. Implications for Low-Order Approximations

The low-order approximations introduced above use the values $H_{2}\left(P\right),H_{3}\left(P\right),\ldots ,H_{r+1}\left(P\right)$. The concentration results show that these values are not interchangeable observations of the same feature of the distribution. Each order weights the distribution differently. The order-two entropy is sensitive to pair collisions and reflects a relatively broad notion of concentration. Higher orders increasingly emphasize repeated observations from the largest probability masses.

This has two consequences. First, adding higher collision orders can improve the extrapolation to Shannon entropy if those orders provide useful information about the shape of the Rényi entropy path near α=1. Second, adding higher orders also shifts the approximation toward statistics that are more sensitive to high-mass events and less sensitive to the tail. In finite samples, higher-order collisions are also harder to estimate, because they require repeated observations of the same state among larger tuples.

Thus the approximation order controls a trade-off. Low-order approximations use statistics that are relatively stable and broadly distributed across the support, but they may retain larger interpolation error. Higher-order approximations can better capture curvature in the Rényi entropy path, but they rely on collision probabilities that are more concentrated and may have higher sampling variability.

## 6. Non-Identifiability from Finite Collision Moments

The preceding sections show how finite collections of collision probabilities can be used to approximate Shannon entropy. We now show that such finite collections cannot, in general, determine Shannon entropy exactly. This provides a formal limitation on any method that uses only finitely many low-order collision moments.

The key point is that collision probabilities are power sums of the probability vector. A sufficiently long sequence of power sums determines a finite distribution up to permutation, but a finite truncation generally leaves degrees of freedom. Along those remaining directions, Shannon entropy can vary.

### 6.1. An Illustrative Example

The simplest example shows that the pair-collision probability alone does not determine entropy. Consider the two distributions $P=\left(\frac{1}{2},\frac{1}{2},0\right)$ and $Q=\left(\frac{2}{3},\frac{1}{6},\frac{1}{6}\right)$. Both are distributions on three symbols. Their pair-collision probabilities are equal:

$$C_{2}\left(P\right)={\left(\frac{1}{2}\right)}^{2}+{\left(\frac{1}{2}\right)}^{2}+0^{2}=\frac{1}{2},$$

while

$$C_{2}\left(Q\right)={\left(\frac{2}{3}\right)}^{2}+{\left(\frac{1}{6}\right)}^{2}+{\left(\frac{1}{6}\right)}^{2}=\frac{4}{9}+\frac{1}{36}+\frac{1}{36}=\frac{1}{2}.$$

Thus the two distributions are indistinguishable by their order-two collision probability.

However, their Shannon entropies differ: H(P)=log2, whereas $H\left(Q\right)=-\frac{2}{3}\;\log\;\frac{2}{3}-2\cdot \frac{1}{6}\;\log\;\frac{1}{6}$. Therefore, knowledge of $C_{2}\left(P\right)$ alone is insufficient to determine H(P).

The same example also shows how higher-order collisions can reveal distinctions hidden from pair collisions. The order-three collision probabilities are

$$C_{3}\left(P\right)={\left(\frac{1}{2}\right)}^{3}+{\left(\frac{1}{2}\right)}^{3}=\frac{1}{4},$$

whereas

$$C_{3}\left(Q\right)={\left(\frac{2}{3}\right)}^{3}+{\left(\frac{1}{6}\right)}^{3}+{\left(\frac{1}{6}\right)}^{3}=\frac{8}{27}+\frac{1}{216}+\frac{1}{216}=\frac{11}{36}.$$

Thus $C_{2}\left(P\right)=C_{2}\left(Q\right)$, but $C_{3}\left(P\right)\neq C_{3}\left(Q\right)$. Pair collisions do not determine triplet collisions, and neither alone determines the full entropy.

### 6.2. General Non-Identifiability

The preceding example is not special. For any fixed number of collision moments there are sufficiently large alphabets on which those moments do not identify Shannon entropy.

**Theorem 2** (Finite collision moments do not determine entropy)**.**
*Let r≥2 and let m≥r+1. Then there exist two distinct probability distributions P and Q on m symbols such that*

$$C_{k}\left(P\right)=C_{k}\left(Q\right),\;k=2,\ldots ,r,$$

*but*

H(P)≠H(Q).

*Consequently, Shannon entropy is not determined by the finite collision sequence*

$$C_{2}\left(P\right),C_{3}\left(P\right),\ldots ,C_{r}\left(P\right)$$

*on the m-symbol simplex when m≥r+1.*

**Proof.**  Let

$$\Delta_{m}^{\circ }=\left\{p\in R^{m}:p_{i}>0,\;\sum_{i=1}^{m}p_{i}=1\right\}$$

denote the interior of the *m*-symbol probability simplex. Define the map

$$F:\Delta_{m}^{\circ }\to R^{r-1}$$

by

$$F\left(p\right)=\left(C_{2}\left(p\right),C_{3}\left(p\right),\ldots ,C_{r}\left(p\right)\right).$$

Equivalently, include the normalization condition and define

$$G\left(p\right)=\left(C_{1}\left(p\right),C_{2}\left(p\right),\ldots ,C_{r}\left(p\right)\right),$$

where

$$C_{1}\left(p\right)=\sum_{i=1}^{m}p_{i}=1.$$

For ℓ=1,…,r, the gradient of $C_{\ell }$ in $R^{m}$ is

$$\nabla C_{\ell }\left(p\right)=\ell \left(p_{1}^{\ell -1},p_{2}^{\ell -1},\ldots ,p_{m}^{\ell -1}\right).$$

Choose a point $p\in \Delta_{m}^{\circ }$ with at least *r* distinct coordinates. Then the matrix with rows

$$\left(p_{1}^{\ell -1},p_{2}^{\ell -1},\ldots ,p_{m}^{\ell -1}\right),\;\ell =1,\ldots ,r,$$

has rank *r* by the Vandermonde rank condition. Hence *G* has rank *r* at *p*.

Because m≥r+1, the level set of *G* through *p* has positive local dimension. By the implicit function theorem, there is a smooth local manifold

$$M=\left\{q\in \Delta_{m}^{\circ }:C_{k}\left(q\right)=C_{k}\left(p\right),\;k=2,\ldots ,r\right\}$$

of dimension at least m−r≥1 near *p*.

It remains to show that Shannon entropy is not constant on this level set. The gradient of Shannon entropy is

$$\nabla H\left(p\right)=-\left(\log p_{1}+1,\ldots ,\log p_{m}+1\right).$$

If *H* were locally constant on M then ∇H(p) would lie in the span of the gradients

$$\nabla C_{1}\left(p\right),\nabla C_{2}\left(p\right),\ldots ,\nabla C_{r}\left(p\right).$$

Equivalently, there would exist constants $a_{0},\ldots ,a_{r-1}$ such that

$$-\left(\log p_{i}+1\right)=a_{0}+a_{1}p_{i}+\cdots +a_{r-1}p_{i}^{r-1},\;i=1,\ldots ,m.$$

That is, the values of −logt−1 at the points $p_{i}$ would agree with a polynomial of degree at most r−1.

Choose *p* with at least r+1 distinct coordinates such that this interpolation relation does not hold. Such a choice is possible because −logt−1 is not a polynomial of degree at most r−1. Therefore,

$$\nabla H\left(p\right)\notin \mathrm{span}\{\nabla C_{1}\left(p\right),\ldots ,\nabla C_{r}\left(p\right)\}.$$

Hence there exists a tangent vector *v* to the level set M at *p* such that

∇H(p)·v≠0.

By the implicit function theorem, there exists a smooth curve p(t)⊂M with p(0)=p and $p^{\prime }\left(0\right)=v$. Along this curve,

$$C_{k}\left(p\left(t\right)\right)=C_{k}\left(p\right),\;k=2,\ldots ,r,$$

but

$${\left.\frac{d}{dt}H\left(p\left(t\right)\right)\right|}_{t=0}=\nabla H\left(p\right)\cdot v\neq 0.$$

Thus, for sufficiently small t≠0, setting Q=p(t) and P=p gives

$$C_{k}\left(P\right)=C_{k}\left(Q\right),\;k=2,\ldots ,r,$$

but

H(P)≠H(Q).

This proves the claim. □

### 6.3. Interpretation of Non-Identifiability

Theorem 2 shows that the approximation error identified earlier is unavoidable in general. A finite collection of low-order collision probabilities defines an equivalence class of distributions. Within that equivalence class, Shannon entropy may vary. Therefore, no estimator or approximation that depends only on $C_{2}\left(P\right),\ldots ,C_{r}\left(P\right)$ can recover H(P) exactly over all distributions on sufficiently large alphabets.

This does not mean that low-order collision approximations are uninformative. Rather, it clarifies what they can and cannot do. They provide finite-resolution summaries of the distribution, and these summaries may approximate Shannon entropy well when the Rényi entropy path is sufficiently regular. But the finite collision sequence does not contain all information needed to determine Shannon entropy in general.

The theorem also explains why increasing sample size alone cannot remove the residual approximation error. Even if the collision probabilities

$$C_{2}\left(P\right),\ldots ,C_{r}\left(P\right)$$

were known exactly they would not generally identify H(P). Additional samples improve estimation of those moments, but additional collision orders are needed to further refine the finite-resolution representation.

### 6.4. Relation to Full Moment Recovery

The non-identifiability result concerns finite truncations of the power-sum sequence. It is compatible with the classical fact that sufficiently many power sums determine a finite distribution up to permutation. For a distribution on *m* symbols, the power sums

$$C_{1}\left(P\right),C_{2}\left(P\right),\ldots ,C_{m}\left(P\right)$$

determine the elementary symmetric polynomials through Newton identities, and hence they determine the multiset

$$\left\{p_{1},\ldots ,p_{m}\right\}$$

up to permutation. Once the probability multiset is determined, Shannon entropy is determined.

Thus the full finite-alphabet distribution can, in principle, be recovered from enough collision moments. The limitation arises when only a fixed low-order truncation is used. The framework developed in this paper sits between these two extremes: it uses a small number of directly estimable collision moments to approximate Shannon quantities while making explicit the residual uncertainty induced by finite moment truncation.

## 7. Numerical Experiments

We now illustrate the behavior of the collision-based approximations in controlled discrete settings. The experiments were designed to separate three effects: approximation error from using finitely many collision orders, estimation error from finite samples, and the concentration behavior induced by increasing collision order. The goal was not to show that collision-based approximations uniformly outperform direct estimators of Shannon entropy or mutual information; rather, the experiments demonstrate the approximation–estimation trade-off developed in the previous sections. Accordingly, the empirical comparisons below are diagnostic: they show how the finite-resolution targets behave relative to Shannon estimators in controlled examples, rather than establishing dominance over existing entropy-estimation methods.

Throughout, logarithms are natural and all entropies are reported in nats. For reference, we include the empirical plug-in estimator and the Miller–Madow correction, both of which target Shannon entropy or mutual information directly. The collision-based approximations instead target finite-resolution functionals constructed from low-order collision statistics. Consequently, differences between the collision curves and the Shannon estimators should not be read only as estimator error; they also reflect the deterministic difference between the finite-resolution target and the Shannon quantity. Note that the subscript *r* on ${\tilde{I}}_{r}$ denotes the number of integer Rényi contrast values used, so the largest collision order is r+1.

### 7.1. Population Approximation Error

We first examined approximation error at the population level, where the joint distribution was known exactly and there was no sampling variability. This isolated the deterministic gap between Shannon mutual information and its finite-order collision approximations.

We began with the binary symmetric channel. We let X∼Bernoulli(1/2), and we let Y=X⊕E, where E∼Bernoulli(ϵ) was independent noise. The joint distribution was

$$P_{XY}=\left(\begin{array}{cc} (1-\epsilon )/2 & \epsilon /2\\ \epsilon /2 & (1-\epsilon )/2 \end{array}\right).$$

Varying ϵ from 0 to 1/2 interpolated between perfect dependence and independence. For each value of ϵ, we computed the Shannon mutual information I(X;Y) and the finite-resolution collision approximations ${\tilde{I}}_{2}$, ${\tilde{I}}_{3}$, and ${\tilde{I}}_{4}$. Because these quantities were evaluated directly from the population distribution, any discrepancy between *I* and ${\tilde{I}}_{r}$ was deterministic approximation error, not sampling error.

**Figure 1:**
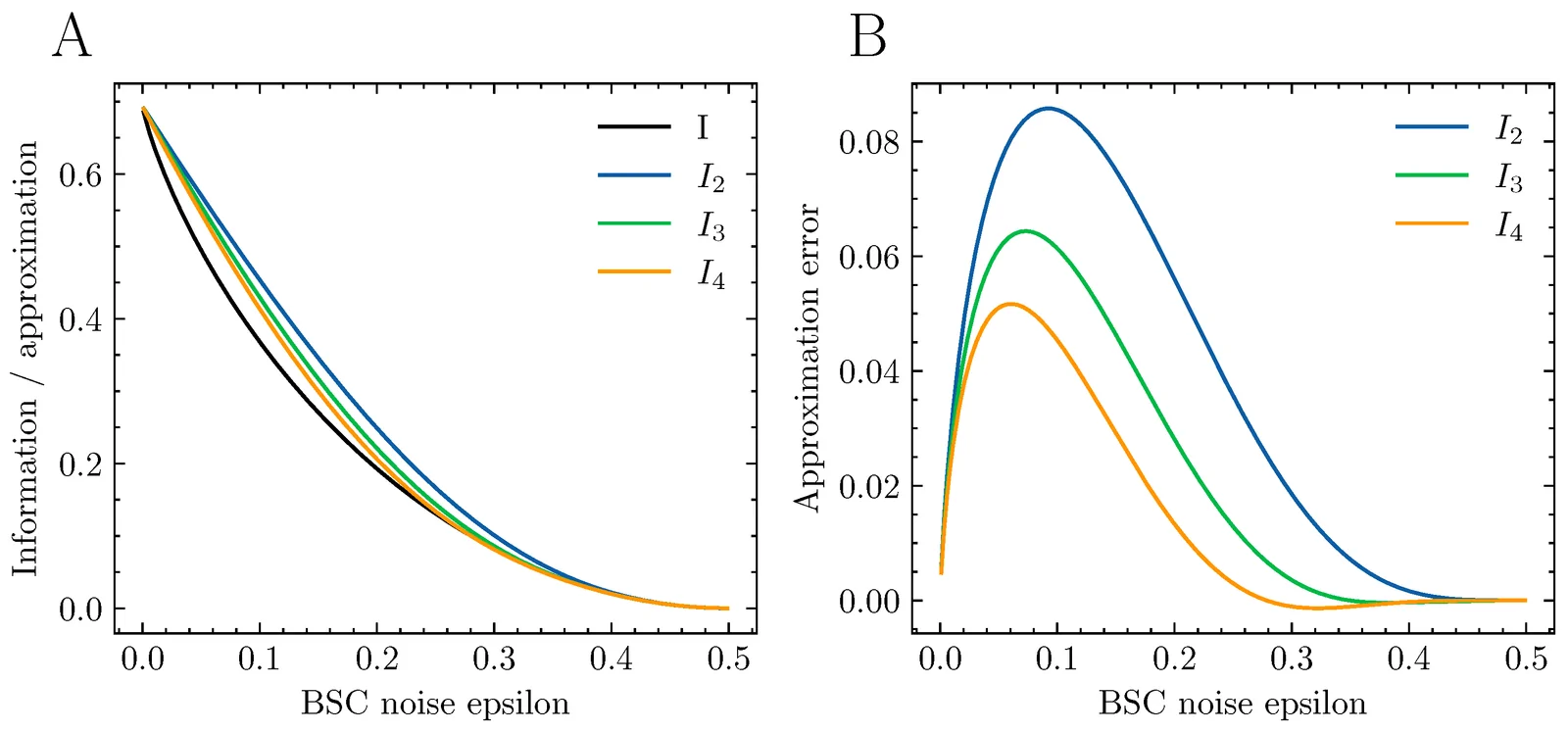
Population approximation error for the binary symmetric channel. (**A**) compares the analytic Shannon mutual information I(X;Y) with finite-resolution collision approximations ${\tilde{I}}_{2}$, ${\tilde{I}}_{3}$, and ${\tilde{I}}_{4}$ as the noise level ϵ varies from near perfect dependence to independence. All approximations match the endpoint behavior but overestimate I(X;Y) at intermediate noise levels. (**B**) shows the corresponding absolute approximation errors. The error is largest at intermediate noise and decreases with approximation order in this example, illustrating the deterministic interpolation error that remains even when the collision probabilities are known exactly.

Figure 1A shows that the collision approximations reproduced the qualitative dependence structure of the binary symmetric channel. All curves decreased from the deterministic limit at ϵ=0 to the independent limit at ϵ=1/2, and the finite-order approximations were exact at both endpoints. For intermediate noise levels, however, the approximations overestimated Shannon mutual information. This discrepancy was not caused by finite samples; it reflected the fact that a finite set of low-order collision statistics captures only a finite-resolution view of the Rényi contrast path.

Figure 1B shows the corresponding absolute errors. The error was small near the endpoints and largest at intermediate noise levels, where the curve relating the integer-order Rényi contrasted to the Shannon point had the greatest effective curvature. Increasing the approximation order reduced the error in this example: ${\tilde{I}}_{2}$ had the largest error, ${\tilde{I}}_{3}$ was smaller, and ${\tilde{I}}_{4}$ was smaller still. Thus, adding collision orders can improve the population approximation, but it does not remove the conceptual distinction between the Shannon target and the finite-resolution target. The remaining gap was a property of the finite-order approximation itself.

### 7.2. Fixed-Order Estimation and the Approximation–Estimation Split

The population experiment above isolated deterministic approximation error. We next added sampling variability to separate approximation error from estimation error. For a fixed collision order *r*, the empirical estimator ${\hat{\tilde{I}}}_{r}$ was computed from finite samples using the without-replacement collision statistics. As the sample size increased, these empirical collision statistics converged to their population collision probabilities, and therefore ${\hat{\tilde{I}}}_{r}$ converged to the finite-resolution population target ${\tilde{I}}_{r}$. Importantly, this target need not equal the Shannon mutual information *I*. Thus increasing *N* removes estimation error for the fixed-order collision approximation, but it does not remove the deterministic approximation error between ${\tilde{I}}_{r}$ and *I*.

We illustrate this distinction using the binary symmetric channel with ϵ=0.2, for which the Shannon mutual information was known analytically. For each sample size *N*, we generated repeated samples from the channel and estimated ${\hat{\tilde{I}}}_{2},{\hat{\tilde{I}}}_{3},{\hat{\tilde{I}}}_{4},{\hat{\tilde{I}}}_{5}$. We compared these estimates with their corresponding population targets ${\tilde{I}}_{2},{\tilde{I}}_{3},{\tilde{I}}_{4},{\tilde{I}}_{5}$, shown as horizontal dotted lines. We also included standard plug-in and Miller–Madow estimators of Shannon mutual information, with the analytic Shannon value shown as a dashed line.

**Figure 2:**
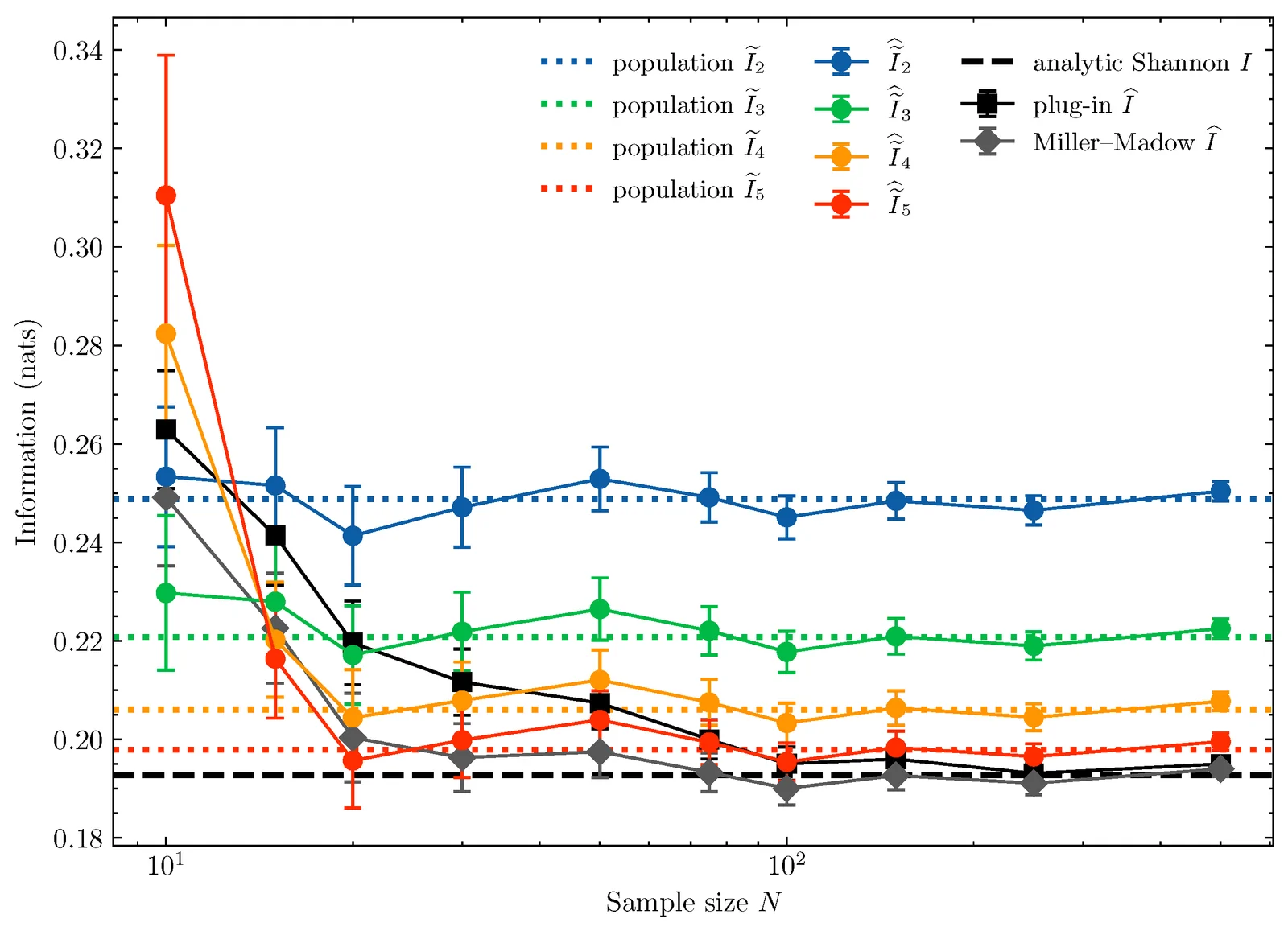
Fixed-order sampling behavior for the binary symmetric channel with ϵ=0.2. For each sample size *N*, repeated samples are drawn from the channel and used to estimate finite-resolution collision approximations ${\hat{\tilde{I}}}_{2},{\hat{\tilde{I}}}_{3},{\hat{\tilde{I}}}_{4},{\hat{\tilde{I}}}_{5}$. Solid colored curves show the empirical means, with error bars indicating two standard errors. Dotted horizontal lines show the corresponding population finite-resolution targets ${\tilde{I}}_{2},{\tilde{I}}_{3},{\tilde{I}}_{4},{\tilde{I}}_{5}$, computed from the exact joint distribution. The dashed black line shows the analytic Shannon mutual information *I*, while the black and gray curves show plug-in and Miller–Madow estimates of *I*. As *N* increases, each collision estimator converges to its own finite-resolution target rather than to the Shannon value. Thus sampling error vanishes with increasing *N*, but the deterministic approximation gap between ${\tilde{I}}_{r}$ and *I* remains for fixed *r*.

Figure 2 shows the expected fixed-order behavior. For each *r*, the empirical collision estimator approached its own population target as *N* increased. The sampling variability, shown by the error bars, decreased with sample size, and the high-order estimators required larger samples before stabilizing. However, the limiting targets remained separated from the analytic Shannon mutual information. The order-two estimate stabilized around ${\tilde{I}}_{2}$, not around *I*; the same pattern held for the higher orders. Increasing the collision order moved the population target closer to *I* in this example, but this behavior need not hold uniformly across distributions and does not make the estimator a conventional Shannon estimator. This illustrates the key approximation–estimation distinction: fixed-order collision methods consistently estimate finite-resolution information targets, while Shannon estimators such as the plug-in and Miller–Madow estimators aim directly at *I*.

The high-order estimates also show the cost of increasing resolution. In this example, larger *r* moved the finite-resolution target closer to the Shannon mutual information, but the corresponding empirical estimator was less stable at small sample sizes. This was expected: higher-order collision statistics depend on rarer coincidence events, so they require more data before their empirical estimates stabilize. Thus increasing collision order can reduce deterministic approximation error while increasing finite-sample estimation error, making the approximation–estimation trade-off visible in the same figure.

### 7.3. Collision Order as a Concentration Filter

The collision order *k* controls which parts of a distribution are emphasized. To illustrate this effect, we constructed categorical distributions with the same support size but different concentration profiles: a uniform distribution and three increasingly tiered distributions in which probability mass was concentrated into progressively smaller high-probability groups. For each distribution, we computed the normalized contribution of each state to the order-*k* collision probability,

$$w_{i}^{\left(k\right)}=\frac{p_{i}^{k}}{\sum_{j}p_{j}^{k}}.$$

These weights identified which states were responsible for the collision statistic $C_{k}\left(P\right)$. At low orders, many states could contribute appreciably. As *k* increased, the powers $p_{i}^{k}$ amplified differences in state probability, shifting the collision statistic toward the highest-probability states.

**Figure 3:**
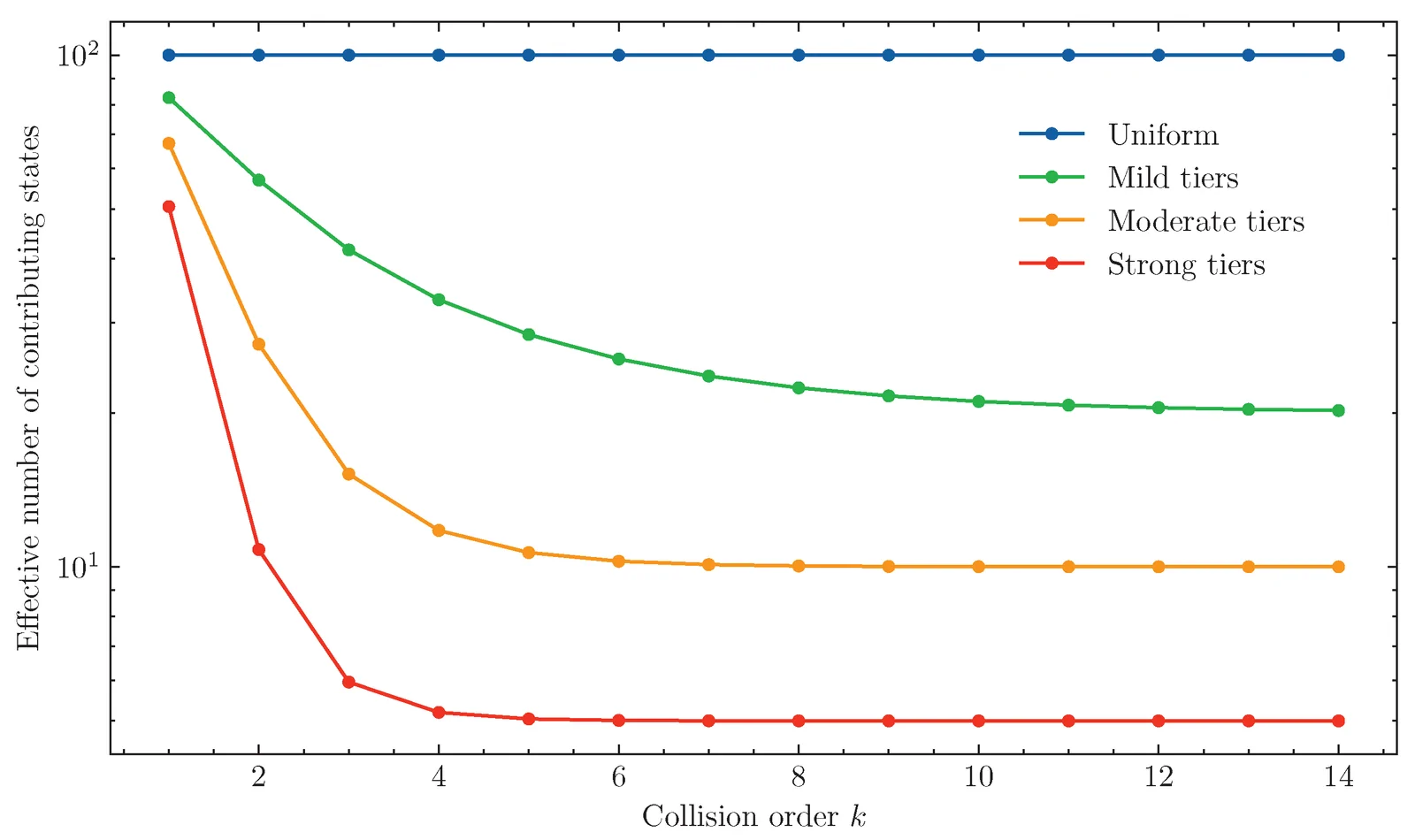
Collision order as a concentration filter. For each distribution, the state-level contribution to the order-*k* collision probability is normalized as $w_{i}^{\left(k\right)}=p_{i}^{k}/\sum_{j}p_{j}^{k}$. The figure shows the effective number of contributing states, $N_{\mathrm{eff}}^{\left(k\right)}=1/\sum_{i}{\left(w_{i}^{\left(k\right)}\right)}^{2}$, as a function of collision order. For the uniform distribution, all states contribute equally and the effective number remains fixed at the support size. For increasingly tiered distributions, the effective number decreases with *k*, showing that higher-order collisions increasingly concentrate on the highest-probability states. Thus collision order changes the resolution of the summary: low orders reflect broad support, while higher orders emphasize dominant mass.

Figure 3 summarizes this shift using the effective number of contributing states,

$$N_{\mathrm{eff}}^{\left(k\right)}=\frac{1}{\sum_{i}{\left(w_{i}^{\left(k\right)}\right)}^{2}}.$$

This quantity was large when many states contributed comparably to $C_{k}\left(P\right)$, and it was small when the statistic was dominated by a few states. For the uniform distribution, the effective number remained equal to the full support size because all states contributed equally at every order. For the tiered distributions, the effective number decreased rapidly with *k*. The strongest tiered distribution collapsed to only a few effective contributors after small increases in collision order, while the milder tiered distributions retained broader support for longer. Thus even moderate collision orders acted as strong concentration filters.

This result clarifies the interpretation of finite-order collision approximations. Adding higher orders does not simply add more information in a neutral way; it changes which parts of the distribution are being probed. Low-order collisions retain broader information about the support, while higher-order collisions preferentially emphasize high-mass states and coherent concentration structure. The order parameter *k* should therefore be understood as a resolution parameter: increasing *k* sharpens the view toward dominant events, but it can also reduce sensitivity to diffuse or low-probability structure.

## 8. Discussion

This paper develops a finite-resolution view of entropy and mutual information based on low-order collision statistics. To be clear, our goal was not to introduce a new universal estimator of Shannon entropy or mutual information, nor to claim that collision-based approximations should always replace direct Shannon estimators. Rather, the framework asks what population-level information is determined by a finite collection of directly estimable coincidence probabilities, how the resulting finite-resolution target differs from the Shannon limit, and what information is necessarily lost when only finitely many collision moments are used. In this sense, the main contribution is to separate the statistical problem of estimating collision moments from the deterministic problem of approximating Shannon entropy or mutual information from a finite set of points on the Rényi entropy path. This separation clarifies the role of sample size. For a fixed collision order, empirical collision probabilities are unbiased *U*-statistics and converge to their corresponding population collision probabilities as the sample size grows. Consequently, the empirical Rényi entropies and their extrapolated quantities converge to finite-resolution population targets. They do not, in general, converge to Shannon entropy or mutual information. The residual difference is not a finite-sample bias; it is deterministic approximation error induced by restricting attention to finitely many low-order collision moments. The interpolation remainder identifies the source of this error in the curvature and higher-order variation of the Rényi entropy path, but it should be interpreted as a structural description rather than as a sharp distribution-free performance guarantee.

The fixed-order asymptotic analysis further shows that the usual Gaussian limit is centered on the finite-resolution target, not on the Shannon quantity. This distinction is important because the sampling behavior of collision statistics depends on the Hoeffding projection structure. Away from degenerate cases, the empirical finite-resolution approximation has the usual $N^{1/2}$-scaled Gaussian fluctuations around its own population target. At uniform distributions, however, the first projection vanishes, and the leading behavior is governed by higher-order Hoeffding components. Thus the asymptotic theory reinforces the central interpretation: collision order determines the population target, while sample size determines how accurately that target is estimated.

For mutual information, the same construction is applied to the marginal and joint distributions through an additive Rényi entropy contrast. This contrast has Shannon mutual information as its order-one limit and vanishes under independence, but for orders other than one it should not be interpreted as an operational or axiomatic Rényi mutual information. It may be negative, and zero contrast need not characterize independence. Its role here is narrower: it provides a collision-based path whose finite-order values can be extrapolated toward the Shannon mutual-information limit.

This perspective also clarifies the role of the numerical experiments. Direct entropy estimators target Shannon entropy or mutual information itself, and more specialized estimators, including Bayesian methods such as NSB, may be preferable when the sole goal is accurate Shannon estimation in sparse regimes [5,20]. The collision-based quantities studied here have a different role. They expose which parts of Shannon information are already visible from low-order coincidence structure, and they make explicit the deterministic approximation error induced by finite moment truncation. In this sense, they should be interpreted less as competitors to direct Shannon estimators than as finite-resolution summaries with transparent population targets and estimable components.

The collision order also provides a structural interpretation of these summaries. As the order increases, collision probabilities become increasingly dominated by the largest probability masses, paralleling the convergence of Rényi entropy to min-entropy [11,15]. Thus the order parameter controls the resolution at which a distribution is viewed: low orders aggregate probability mass broadly, while higher orders emphasize repeated observations from high-probability states. This provides a formal basis for interpreting low-order collision approximations as scale-dependent or resolution-dependent summaries of uncertainty.

The non-identifiability result places a limit on what such summaries can accomplish. A finite sequence of collision moments generally does not determine Shannon entropy. Even if the collision probabilities $C_{2}\left(P\right),\ldots ,C_{r}\left(P\right)$ were known exactly, multiple distributions can share those values while having different entropies. This shows that the approximation error is not an artifact of a particular estimator. It is intrinsic to representing a distribution through finitely many low-order power sums. At the same time, the classical relationship between power sums and finite multisets shows that sufficiently many moments can determine a finite distribution up to permutation [21]. The framework studied here occupies the intermediate regime: it uses only a small number of collision moments, accepting finite-resolution approximation in exchange for direct estimability and interpretability.

One natural application is the comparison of clusterings. Given two partitions of the same elements, the contingency table between cluster labels defines a joint empirical distribution, so standard information-theoretic clustering similarities, including mutual information, normalized mutual information, adjusted mutual information, and variation of information, can be written as functions of this joint distribution and its marginals [22,23,24]. Pair-counting measures such as the Rand index and adjusted Rand index instead depend on whether pairs of elements are co-assigned under the two partitions [25,26]. Collision probabilities provide a direct bridge between these views: pair-counting corresponds to order-two collision structure, while higher-order collisions measure agreement among triples, quadruples, and larger tuples [27]. This connection complements prior work showing that clustering comparisons can be sensitive to random models and structural biases [24], as well as element-centric approaches that compare clusterings through the relationships induced around individual elements [28]. The present paper does not develop a clustering similarity measure directly; instead, it provides the information-theoretic substrate for a related idea: clustering similarities based on pairs, triples, and higher-order tuples can be viewed as finite-resolution points on a collision-information path. This suggests a principled way to interpolate between pair-counting and information-theoretic comparisons, while also clarifying the limits of any fixed-order tuple statistic.

The collision perspective also connects naturally to diversity measures in ecology and related fields. The order-two collision probability $C_{2}\left(P\right)=\sum_{i}p_{i}^{2}$ is the probability that two independent draws belong to the same category, and its complement is Simpson’s diversity index [13]. The reciprocal $1/C_{2}\left(P\right)$ is commonly interpreted as an effective number of types and corresponds to the order-two Hill number [14]. Thus, the pairwise collision entropy $H_{2}\left(P\right)$ is closely related to established diversity measures: it is the logarithm of inverse Simpson concentration. From this perspective, our framework extends the logic of pairwise diversity from two-sample coincidences to a hierarchy of higher-order coincidence events, and it asks how these finite-order diversity summaries approximate Shannon entropy.

Several limitations remain. First, the interpolation bounds depend on derivatives of the Rényi entropy or contrast path. These bounds are useful conceptually, but sharper distribution-dependent bounds would make the approximation theory more predictive. Second, higher collision orders introduce a bias–variance trade-off. They may reduce interpolation error by capturing more of the local shape of the Rényi path, but they also rely on rarer coincidence events and can be harder to estimate in finite samples. Third, the Rényi entropy contrast used for the mutual-information approximation is not a full Rényi mutual information in the axiomatic or operational sense. Other generalizations, such as Sibson and Arimoto mutual information, satisfy different properties and serve different purposes [17,18,19]. Here, the contrast is used because it arises naturally from combining marginal and joint collision entropies and because it has the Shannon mutual information as its order-one limit.

Future work could proceed in several directions. One direction would be to develop sharper approximation bounds for specific distribution classes, such as heavy-tailed distributions, near-uniform distributions, or structured joint distributions. A related direction would be to study collision-based finite-resolution approximations for block entropy in dependent symbolic sources, including long-range dependent or heavy-tailed processes. In such settings, the effective alphabet grows rapidly with block length, making direct Shannon estimation difficult and making low-order collision structure potentially informative [29,30,31]. A third direction would be to study adaptive schemes that choose collision orders based on sample size and observed concentration. A fourth direction would be to connect the finite-resolution collision framework to hypothesis testing, where low-order collision contrasts may provide simple statistics for detecting particular forms of dependence. Finally, the clustering setting suggests a broader program of tuple-order similarity measures that explicitly control the resolution at which two partitions are compared.

A further research direction would be to characterize the tasks for which the additive Rényi entropy contrast used here is equivalent, or approximately equivalent, to Shannon mutual information. Because $K_{\alpha }$ is not an operational Rényi mutual information for α≠1, such equivalence should not be assumed in general. However, there may be specific estimation, ranking, testing, or model-comparison settings in which replacing Shannon mutual information by a finite-order collision contrast preserves the relevant ordering or decision. Identifying these settings would clarify when collision-based finite-resolution summaries can serve as principled proxies for Shannon information and when they should instead be interpreted only as distinct resolution-dependent summaries.

Overall, the collision perspective provides a simple way to separate what is statistically estimable from what is informationally identifiable. Low-order collision statistics are not sufficient to determine Shannon entropy in general, nor are they intended to replace direct estimators of Shannon information. Their value is that they define a transparent hierarchy of finite-resolution information summaries. This hierarchy links coincidence counts, Rényi entropy, Shannon limits, and mutual information within a single approximation–estimation framework.

## Data Availability

No empirical datasets were analyzed. The numerical experiments use synthetic distributions generated from the specifications described in the text. Code for reproducing the figures is available at https://github.com/ConnectedDataHub/collision-information (accessed on 24 July 2026).

## Appendix A. Hoeffding Projections and Degenerate Collision Limits

### Appendix A.1. First-Order Projection and Joint Covariance

For the kernel $h_{k}\left(x_{1},\ldots ,x_{k}\right)=1\left\{x_{1}=\cdots =x_{k}\right\}$, the first projection is

$$\psi_{k}\left(x\right)=E\;\left[h_{k}\left(x,X_{2},\ldots ,X_{k}\right)\right]-C_{k}\left(P\right)=p_{x}^{k-1}-C_{k}\left(P\right).$$

Therefore,

$$E\;\left[\psi_{k}\left(X\right)\psi_{\ell }\left(X\right)\right]=\sum_{x}p_{x}p_{x}^{k-1}p_{x}^{\ell -1}-C_{k}\left(P\right)C_{\ell }\left(P\right)=C_{k+\ell -1}\left(P\right)-C_{k}\left(P\right)C_{\ell }\left(P\right).$$

Since the order-*k* and order-*ℓU*-statistics have first-order coefficients *k* and *ℓ*, respectively, their asymptotic covariance is

$$k\ell \left[C_{k+\ell -1}\left(P\right)-C_{k}\left(P\right)C_{\ell }\left(P\right)\right].$$

### Appendix A.2. Uniform-Distribution Degeneracy

Suppose *P* is uniform on *m* states. Then $p_{x}=1/m$ and $C_{k}\left(P\right)=m^{1-k}$, so the first projection vanishes, $\psi_{k}\left(x\right)=p_{x}^{k-1}-C_{k}\left(P\right)=0$. The second projection is

$$\psi_{k,2}\left(x,y\right)=E\;\left[h_{k}\left(x,y,X_{3},\ldots ,X_{k}\right)\right]-C_{k}\left(P\right)=m^{-(k-2)}\left[1\left\{x=y\right\}-\frac{1}{m}\right].$$

The associated integral operator has eigenvalue $m^{-(k-1)}$ with multiplicity m−1 on the mean-zero subspace. Degenerate *U*-statistic theory therefore gives

NC^k(P)−m1−k⇒k2m−(k−1)∑s=1m−1(Zs2−1),

where $Z_{1},\ldots ,Z_{m-1}$ are independent standard normal variables. Since

$$\sum_{s=1}^{m-1}Z_{s}^{2}∼\chi_{m-1}^{2},$$

this is equivalently

NC^k(P)−m1−k⇒k2m−(k−1)χm−12−(m−1).

Applying the delta method to $H_{k}={\left(1-k\right)}^{-1}\log C_{k}$ at $C_{k}=m^{1-k}$ gives

$$N\left[{\hat{H}}_{k}\left(P\right)-\log m\right]\Rightarrow -\frac{k}{2}\left[\chi_{m-1}^{2}-\left(m-1\right)\right].$$

### Appendix A.3. Joint Covariance for Marginal and Joint Collision Statistics

For paired observations Z=(X,Y), define

$$\psi_{A,k}\left(Z\right)=\left\{\begin{array}{cc} p_{X}{\left(X\right)}^{k-1}-C_{k}\left(X\right), & A=X,\\ p_{Y}{\left(Y\right)}^{k-1}-C_{k}\left(Y\right), & A=Y,\\ p_{XY}{\left(X,Y\right)}^{k-1}-C_{k}\left(X,Y\right), & A=XY. \end{array}\right.$$

Then the first-order covariance between the order-*k* collision statistic for *A* and the order-*ℓ* collision statistic for *B* is

$$k\ell \;E\;\left[\psi_{A,k}\left(Z\right)\psi_{B,\ell }\left(Z\right)\right].$$

This yields the covariance matrix used in Equation (5).
